# Properties of FRC with Carbon Fibres from Recycled Wind Turbine Blades

**DOI:** 10.3390/polym17233199

**Published:** 2025-11-30

**Authors:** Filip Szmatuła, Jacek Korentz

**Affiliations:** Institute of Civil Engineering, University of Zielona Gora, Prof. Z. Szafrana 1, 65-417 Zielona Góra, Poland

**Keywords:** fibre reinforced concrete, recycled carbon fibre, mechanical properties

## Abstract

This paper investigates the use of recycled carbon fibre (rCF) from wind turbine blades in fibre-reinforced concrete (FRC). The research demonstrates the combined effects of fibre length (25 mm, 35 mm, and 45 mm) and fibre content (0.29%, 0.58%, and 0.87% by volume). The experimental programme included the investigation of compressive and tensile splitting strengths, as well as the determination of the Brittleness Index and fracture energy. The transfer of tensile forces through fibres was assessed based on the surface area of split samples. Tensile strength was determined for two loading directions: parallel and perpendicular to the direction of concreting. It was found that the maximum fibre addition reduced the compressive strength by up to 9% and that the tensile strength was significantly dependent on the fibre orientation, which was determined by the direction of concreting. The tensile strength perpendicular to the direction of concreting increased by a maximum of 11.8% and parallel to the direction of concreting by a maximum of 66% compared to plain concrete, depending on the fibre content and length. The research also demonstrated the synergy of pull-out and rupture of fibres in transmitting tensile forces. These studies provide important insights into the applicability of rCF as a dispersed reinforcement in concrete and have important implications for sustainability in the construction sector.

## 1. Introduction

Different types of fibres are now being used in the construction industry to improve the properties of concrete, primarily its ductility and flexural as well as tensile strength. Steel fibre is the most recognized among the commonly used types of fibres. However, carbon fibre’s higher tensile strength, low density, and corrosion resistance make it a potentially better alternative to steel fibre, despite its higher cost. Currently, carbon fibres are mainly used in the construction sector for repair, renovation, and structural strengthening works. The application of carbon fibre as dispersed reinforcement is nowadays limited and is still under development.

Activities are also being undertaken on the use of recycled carbon fibres obtained, among other sources, from the recycling of wind turbine blades. Undoubtedly, wind energy is one of the most important renewable energy sources, and the first wind turbines have already reached the end of their service life. Therefore, the biggest challenge for wind power in the near future will be blade recycling.

Turbine blade recycling aligns with the circular economy, a key pillar of the European Union’s Green Deal. In the context of wind energy, this involves not only the production of green energy but also a commitment to managing the entire turbine lifecycle responsibly. One example of this commitment is the use of recycled carbon fibres as reinforcement in concrete elements. This innovative approach reduces waste and creates new, valuable products.

### 1.1. Recycling of FRP Composites

Wind turbine (WT) blades are primarily made of composite materials composed of thermoset resin and glass fibres (GFs) or carbon fibres (CFs), referred to as glass or carbon fibre-reinforced polymer (GFRP or CFRP, respectively). Currently, CFs are mainly used in girders or structural components of wind blades longer than 45 m in both terrestrial and marine systems. The content of CFRP in the smallest wind turbine blades is equal to only 9%, but it increases to 55% for larger blades when their length exceeds 70 m [1].

The structural part of the blade is composed of GFRP or CFRP, supplemented with lightweight filler materials such as balsa or polyurethane foam [2]. Namely, between 80% and 90% of the blade’s mass is composed of composite material, with 60% to 70% consisting of reinforcing fibres and the remaining 30% to 40% made up of resin. The remaining 10% to 20% of the blade’s mass consists of wood, foam, balsa, adhesives, gel coat, and paint [3]. It is noteworthy that the first wind turbines are approaching the end of their useful life, which is typically 20 to 25 years [4]. It is estimated that about 25,000 tons of blades will reach the end of their life annually, as of 2025 [5]. Finally, 2 million tons of wind turbine blade waste will be generated by 2050 [6].

There are three methods for utilizing fibre-reinforced polymer, FRP, waste: storage, incineration and co-incineration, and recycling. However, recycling is the most desirable method [7]. Nevertheless, the recycling of wind turbine blades is a complex and costly process, as glass or carbon composites are difficult to process and require specialized equipment and technology. Used wind turbine blades can also be repaired and rehabilitated for reuse in wind turbines [8], or they can be repurposed for alternative applications, such as small architectural objects, pedestrian overpasses, building elements, and noise barriers [9].

Unfortunately, only a part of FRP waste is recycled, with the most common recycling methods including mechanical, thermal, and chemical recycling, as well as high-voltage fragmentation [7]. The result of these recycling processes is matrix degradation, yielding composite wastes that can be reused, including oils, gases, and solid products like fibres, shavings, and dust [10,11,12].

Research into the use of recycled fibre-reinforced polymers in cementitious materials in the construction sector is multifaceted. Appropriately fragmented glass fibre composite waste can be added into a cement kiln as a raw material for cement clinker production and as a fuel [13]. On the other hand, shredded FRP composite wastes, depending on the fraction sizes obtained after grinding, are being examined as a substitute for cement, fine aggregate, and coarse aggregate replacement, as well as dispersed reinforcement in the cement matrix [14,15,16].

### 1.2. FRP Fibres in Concrete

Fibre-reinforced concrete (FRC) offers significant economic and environmental benefits, including a reduced carbon footprint, conservation of natural resources, increased recyclability, and the potential of utilizing waste materials [17].

FRC is a constantly evolving technology that is competitive in many applications. FRC is a cement composite with dispersed reinforcement in the form of steel fibres or non-metallic fibres. These include fibres of varying moduli of elasticity and that are high-strength, such as carbon, polyvinyl chloride, steel, asbestos, and glass fibres, which can significantly increase the strength of concrete, or low-strength, such as nylon, acrylic, and polypropylene fibres, which mainly improve ductility and minimize cracking [18].

Currently, non-metallic fibres can be competitive with steel fibres despite their low Young’s modulus (except for carbon fibres). This is due to their different mechanism of pulling out of the matrix and the increased number of individual fibres per unit volume compared to steel fibres. Based on length, a distinction is made between macrofibres and microfibres. Longer fibres can transmit loads acting on structures, so they can perform a structural function and thus replace traditional bar reinforcement. Shorter fibres, as a result of their large number even at low dosages, are more effective in bridging microcracks and reducing the formation of shrinkage cracks [19].

In the search for optimal FRC performance, various fibre combinations are being studied, for example, steel and polyester fibres [20], steel and polypropylene fibres [21], steel and basalt fibres [22], steel, polypropylene, and Kevlar fibres [23], as well as combinations of fibres with reinforcement [24]. The hybridization of different fibre types can improve the strength properties of hybrid fibre-reinforced concrete (HFRC) and provide positive synergistic effects [25]. Combining fibres of different lengths, diameters, moduli of elasticity, and tensile strength is one of several hybridization strategies [18]. Above that, a combination of short and long fibres provides better results than single-length fibres. The issue stems from the use of well-sorted aggregate [26].

Dispersed reinforcement primarily increases tensile strength and ductility, reduces shrinkage from drying, increases impact strength, and, above all, increases the energy dissipated during crack propagation in concrete. The mechanical properties of FRC depend on the type of fibres, their shape, length, diameter, and, most importantly, their quantity. Unlike conventional reinforcing bars, which are specially designed and placed in the tensile zone of the elements, fibres are thin and short, and their distribution in the concrete element is random. The random fibre distribution makes FRC an anisotropic material, which in practice requires ensuring optimal fibre orientation for different load directions [27]. The orientation of the fibres depends on several variables: the wall effect, the casting and compaction process, the concrete consistency, and the types and amounts of fibres [28]. Fibres in the concrete mix tend toward sedimentation and agglomeration and to be self-levelling [29,30].

In self-compacting concretes (SCCs), steel fibres tend to orient in the direction of the mix flow [31]. The orientation of the fibres perpendicular to the direction of concreting is particularly important when the fibres need to carry stresses from a specific load direction [32]. Fibre in FRC works differently in monolithic retaining walls and columns than in slabs or concrete floors, where concreting is in the same direction. Therefore, it is necessary to search for a set of engineering tools to predict fibre orientation in FRC [30]. FRC tests with steel fibres [33] showed that the splitting tensile strength parallel to the direction of concreting was up to 1.5 times higher than for samples tested perpendicular to the direction of concreting. Similar observations were noted in the study in [34], which showed an enhancement in tensile strength of up to 33% and a nearly fourfold increase in dissipated energy. Therefore, to characterize the properties of FRC used in structural elements, there is a need for samples that take into consideration the fibre orientation and can simulate the actual fibre reinforcement mechanisms present in the real structural element [35].

### 1.3. Carbon FRP Fibres in Concrete

Nowadays, research is being conducted on the use of virgin carbon fibre (vCF) and thermally or chemically recycled carbon fibre (rCF) as fibre reinforcements in concrete (CFRC).

When choosing the type of carbon fibre, economic and environmental aspects are also important. The cost of recycled carbon fibre, rCF, is about 15% of the cost of producing virgin carbon fibre, vCF [36]. In addition, rCF recycling consumes only 5% of the energy required to produce vCF [37]. Therefore, the commercialization of rCFs is warranted due to their economic and sustainability advantages. However, it is also necessary to confirm the technical benefits. The mechanical properties of rCF are comparable to those of vCF, allowing for the substitution of virgin fibres with recycled fibres, thereby ensuring the previously noted advantages. The best quality of rCF is obtained from thermal and chemical recycling methods, while the worst quality is obtained from mechanical methods [38]. However, it must be mentioned that the mechanical properties of pyrolytic fibres can deteriorate as a result of recycling by thermal methods. Tests conducted on carbon fibres show that the pyrolysis process can leave about 2% of the matrix on the fibre surface, and as a result, the tensile strength of rCF decreases by about 20% compared to vCF [39]. On the other hand, in the case of microwave pyrolysis, the tensile strength of rCF is about 99.4% of that of vFC with a fibre recovery rate of 96–98% [40].

The available research results mostly show an improvement in the mechanical properties of concrete with the addition of vCF. Nevertheless, the results of these studies often differ. Namely, studies of CFRC with the addition of vCF [41,42,43] showed an increase in compressive strength from 25% to 50%, depending on fibre content. On the other hand, studies [44,45] showed a slight increase in compressive strength by a maximum of 10%. In the case of flexural strength, depending on fibre content, an increase ranging from 15% to 50% was observed [41,44], and in the case of splitting tensile strength, the enhancement varied from 10% to 130% [41,44,46]. The increases in strength also depended on fibre content.

The use of carbon fibres in special concretes is also being studied. Adding vCF fibres to self-compacting concrete increases compressive strength by 47.6%, splitting tensile strength by 27.6%, and flexural strength by 67.2% [47]. Carbon fibre-modified vCF polymer concretes increase flexural strength and splitting tensile strength by 36% and 61%, respectively, depending on the ratio of polymer and cement [48]. In previous concretes, properly prepared, highly comminuted vCF resulted in an improved workability and infiltration rate, as well as an increase in compressive and tensile strength and Young’s modulus [49].

In conclusion, studies on the impact of vCF on the CFRC mechanical properties have shown varying results, due to the use of different fibre dosages with distinct physical, mechanical and geometric properties, various types of cement, aggregates, water-to-cement ratios, and workability requirements. In addition, the properties of CFRC are influenced by the shape of the fibre cross-section [50], the relationship between the length and diameter of the fibre aspect ratio [51], as well as the consistency of the mixture [45] and the mixing procedure [52].

Moreover, there are limited studies on the use of rCF in cementitious composites. A study in [53] of ultrahigh-performance concrete (UHPC) with the addition of very short (1.5 mm) rCF showed that the tensile strength of the rCF specimens (36 kg/m^3^) was 32% higher, and the flexural strength was 9% lower than that of the reference specimens. Test results [54] of cement mortars with rCF from the automotive industry indicate satisfactory increases in compressive and flexural strength in the range of 10–18% for specimens with a fibre dosage of about 4% and length of 2–5 mm. On the other hand, according to the study in [55], the addition of rCFs with lengths of 3, 6, and 20 mm to cement mortars from mechanically recycled CFRP panels resulted in a slight decrease in compressive strength, an increase in flexural strength of up to 20% and a very large increase in fracture energy of up to 6000%. In the study in [16], on the effect of different amounts and sizes of rCF pieces from CFRP crushing on CFRC strength, a very interesting conclusion was drawn. Namely, in addition to changes in compressive strength from −10% to +20% and flexural strength from −20% to 32%, there was a significant effect of the ratio of rCF piece size to specimen size (size ratio). Tests showed an increase in compressive and flexural strength with a decreasing size ratio.

Carbon fibres have thermal and electrical conductivity similar to metals. Moreover, they are characterized by thermostability and corrosion resistance. Under the influence of load, temperature, and other external factors, CFRC will exhibit pressure and temperature sensitivity with a change in CF spacing, which is one of its most unique attributes. Therefore, as a functional filler, CFs have significant advantages in the manufacturing of smart concrete [56].

There is limited research on rCF derived from recycled wind turbine blades and its application in CFRC. Therefore, it is essential to investigate its behaviour in CFRC and compare it with other commercially available fibre types to assess its suitability as reinforcement for concrete. This study fills a research gap in the application of rFC derived from recycled wind turbine blades in cementitious composites. To this end, selected mechanical and physical properties of CFRC fibres were examined. Specifically, this study investigated how the quantity, length, and orientation of rCF fibres affect the compressive strength, splitting tensile strength, Brittleness Index, energy dissipation, and apparent density of concrete.

## 2. Materials and Methods

**Figure 1:**
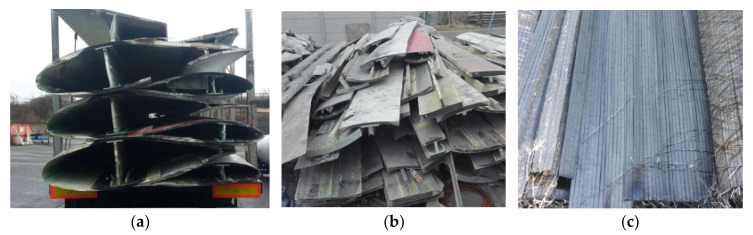
Steps in recycling wind turbine blades: (**a**) transportation, (**b**) preparation, (**c**) recycling.

In the study, carbon fibres from recycled wind turbine blades made with carbon composites were used. The carbon fibres were recovered by pyrolysis according to Anmet’s technology [57] (Przedsiębiorstwo Wielobranżowe ANMET Andrzej Adamcio, 67-300 Wiechlice, Poland). This process can be used when the propellers, reinforced with fibres, have a length of not less than 50 m. Strips about 50 cm wide (Figure 1b) are cut from the prepared pieces (Figure 1a). The thickness of the blade plating varies from about 10 cm near the hub to 2–3 mm at the end of the blade.

**Figure 2:**
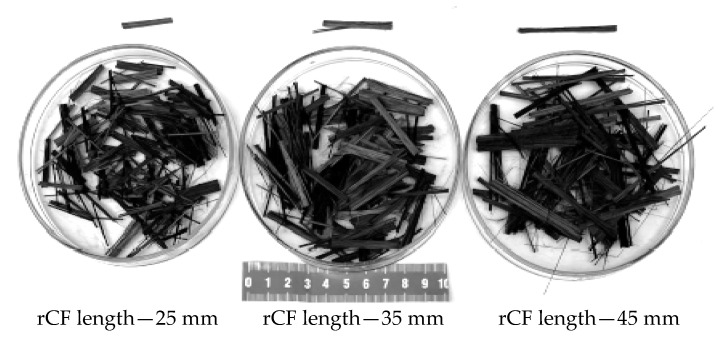
Dispersed reinforcement made of rCF.

The cut strips require cleaning by eliminating glue residues and other composite additives. The finished strips are cut into segments of about 170 cm in length. The next step is the pyrolysis process. The strips are subjected to a temperature of 500 °C for 60 min. After this process, the fibres contain charred resin in an amount equal to about several percent of the total recycled content. The fibre strips are then subjected to rolling (fibrillation), which results in the formation of fibre strands that are about 10 mm wide (Figure 1c), and 0.10 to 0.35 mm thick, with a fibre diameter of 6 μm. Rolling makes it possible to soften the fibres without distorting the parallel arrangement of the internal structure. Subsequently, the prepared fibres are subjected to an oxygen heating process at 500 °C for 30 min. Reheating the strips removes the charred resin, resulting in carbon fibres with a purity close to 100%. The next step is to cut the strips with guillotines into fibres of appropriate length (Figure 2). The average density of the obtained fibres is 1.724 g/cm^3^, and their average tensile strength is equal to 1650 MPa.

For the purpose of this study, fibre strands measuring 10 mm in width were cut into lengths of 25 mm, 35 mm, and 45 mm (Figure 2). The cut resulted in their defragmentation and division into narrower strands.

The experimental campaign involved the determination of the compressive strength fcc, and splitting tensile strength fct of three test series CF-25, CF-35, and CF-45 with carbon fibre lengths of 25 mm, 35 mm, and 45 mm, and with different volumetric contents of 0.29% (5.0 kg/m^3^ by weight), 0.58% (10.0 kg/m^3^ by weight), and 0.87% (15.0 kg/m^3^ by weight).

The concrete was designed assuming a strength class of C35/45. Portland cement CEM-I 42.5R from the Górażdże cement plant and natural aggregate with fractions of 0/2 mm and 2/8 mm were used. Table 1 shows the composition of the reference concrete mixture C-0 without fibres and concrete mixes with the addition of various rCF dosages. The water–cement ratio *w*/*c* for all mixes was 0.60.

Table 2 shows the results of workability tests carried out by the slump testing method according to [58] for the C-0 reference mix, as well as the CF-25, CF-35, and CF-45 mixes with fibres of three lengths (25 mm, 35 mm, and 45 mm), and four dosages (0.29%, 0.58%, 0.87%, and 1.16%). The workability class was characterized according to the standard [59]. The C-0 reference concrete mix is classified as S3, while all mixes with fibre additions were classified as S1.

From the results presented in Table 2, it can be seen that concretes with rCFs had high water demands compared to plain concretes, thus causing significant deterioration in the workability. According to [59], all concretes with rCFs were characterized by the consistency class S1. As can be seen, the workability of the concrete deteriorated with an increase in the number of carbon fibres. The cone slump decreased from 30 mm for a fibre content equal to 0.29% to 10 mm for a fibre content of 0.87%. This is in accordance with other studies in which the slump also decreased, and thus, the concrete mixture’s workability deteriorated with an increase in the number of carbon fibres [46] and of steel fibres [60].

**Figure 3:**
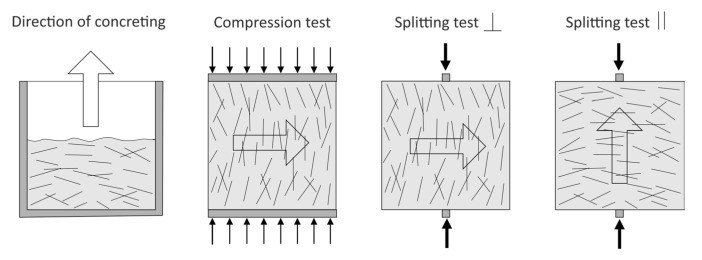
FRC strength test methods.

Regarding the compressive strength tests, they were carried out perpendicular to the direction of concreting, while splitting tensile strength tests were conducted both perpendicular and parallel to the direction of concreting (Figure 3).

**Figure 4:**
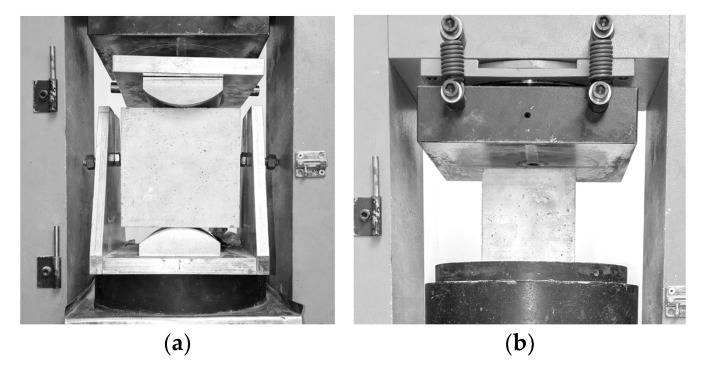
Test machine setup: (**a**) Splitting tensile strength, (**b**) compressive strength.

The setup of the machine for testing tensile strength in splitting and compressive strength is shown in Figure 4.

Compressive strength tests were performed on cubic specimens with a side length of 100 mm according to [61], and splitting tensile strength tests were carried out on cubic specimens with a side length of 150 mm according to [62].

## 3. Results and Discussion

The results of this study allowed for understanding the modification of some mechanical and physical properties of concrete with the addition of rCFs, compared to plain concrete, taking into account the following effects: 1—the orientation of the rCFs; 2—the content of rCF; and 3—the length of the rCFs.

Table 3 summarizes the average measured values obtained from the tests, where $f_{cc}$ is the compressive strength, $f_{ct,⊥}$ is the splitting tensile strength of samples tested perpendicular to the direction of concreting, and $f_{ct,∥}$ is the splitting tensile strength of samples tested parallel to the direction of concreting, and the calculated values, where *BI* is the Brittleness Index, ${FE}_{⊥}$ is the fracture energy of samples tested perpendicular to the direction of concreting, and ${FE}_{∥}$ is the fracture energy of samples tested parallel to the direction of concreting.

Analysis of the obtained compressive and splitting tensile strength results was carried out using the Strength Enhancement Index [63] calculated according to Formula (1):

(1) $$Strength\;Enhancement=\frac{Strength\;with\;fibres\;-Strength\;without\;fibres}{Stength\;without\;fibres}\;(\%)$$

The analysis of the Brittleness Index *BI* and fracture energy *E* was conducted analogously.

### 3.1. Apparent Density

**Figure 5:**
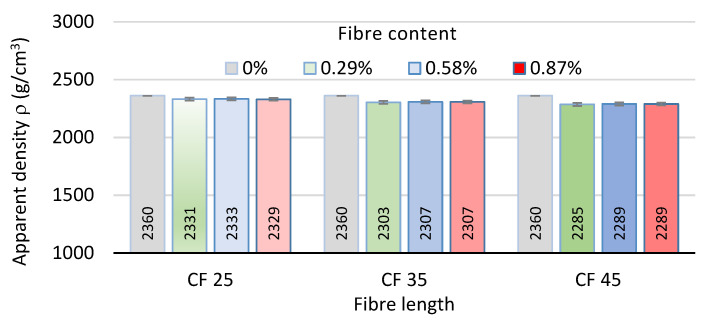
Effect of rCF addition on the concrete apparent density.

Figure 5 shows the results of testing the effect of rCF length and amount on the concrete apparent density ρ. The apparent density of the specimens without the addition of fibres was equal to 2360 kg/m^3^.

As expected, the addition of rCFs caused a decrease in apparent density proportional to the amount of added fibres. The maximum decrease in density was equal to 3% and was denoted for concrete with a fibre content of 1.16%. It was concluded that this was a result of the combined effect of the addition of fibres of low specific mass and an increase in the porosity of the concrete.

### 3.2. Compression

**Figure 6:**
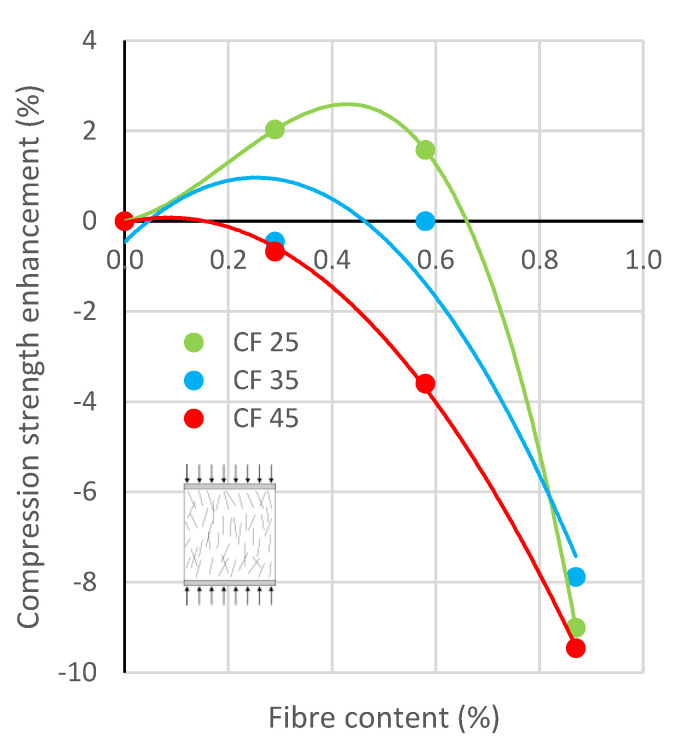
Effect of rCF content and length on concrete compressive strength *f*_cc_.

Figure 6 shows graphs illustrating the changes in compressive strength of concrete *f*_cc_ with the addition of rCFs. The average compressive strength of concrete without fibres was equal to 44.4 MPa, and the effect of rCF addition on compressive strength varied depending on the fibre length and dosage. Fibre additions of 0.29% and 0.58% resulted in strength changes of no more than four percent, with a slight increase in strength for CF-25 specimens, and a slight decrease in strength for specimens reinforced with longer fibres—CF-35 and CF-45. In contrast, the addition of 0.87% of fibres, regardless of their length, led to a decrease in compressive strength of nearly 10%.

A similar trend of decreasing compressive strength with increasing fibre content was noted in the study in [64], in which, for carbon fibre content equal to 0.2%, 0.4%, 0.6%, and 0.8%, the compressive strength of samples changed by 4.93%, 15.02%, −15.02%, and −23.74%, respectively, compared to the specimen without fibres.

The observed decreases in the compressive strength were primarily the result of the standard requirements for performing the compression test. According to the standard [61], the trowelled surface of the specimen (top surface created during casting) cannot have contact with the pressure plates of the compression testing machine, and thus has to be the side surface during testing. Therefore, the compressive force acted perpendicular to the direction of concreting and, as a result, parallel to the orientation of the rCFs (see Figure 3), which weakened the specimen in the transverse direction. This was mainly the effect of the smooth surface of the used fibres, since the resin, as a coupling agent which is an inorganic compound, reduced the adhesion between the fibres and the cement paste.

### 3.3. Tension

Splitting tensile tests were conducted parallel ($f_{ct,∥}$) and perpendicular ($f_{ct,⊥}$) to the direction of concreting. The tensile splitting strength *f_ct_* was calculated using the following formula:

(2) $$f_{ct}=\frac{2F}{\pi Lh}$$

where *F* is the maximum load, *L* is the length of the line of contact of the specimen, and *h* is the designated cross-sectional dimension.

**Figure 7:**
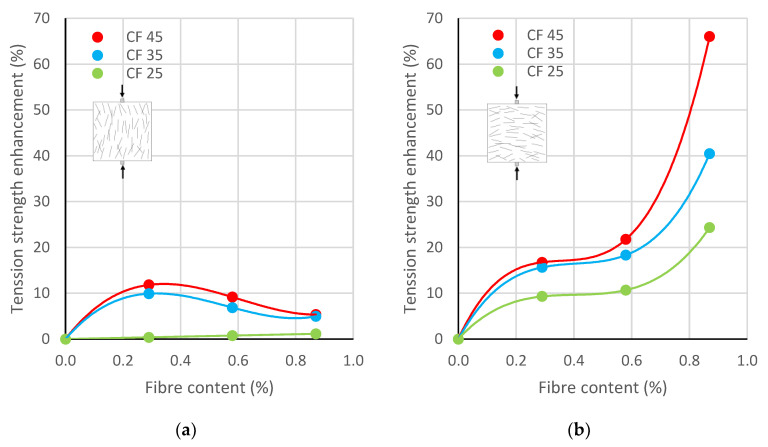
Tensile strength enhancement vs. rCF content for different fibre lengths: (**a**) Perpendicular to the direction of concreting, (**b**) parallel to the direction of concreting.

The results of tensile tests are given in Figure 7. The tensile strength of the concrete without fibre addition was equal to 2.62 MPa. While decreases in compressive strength had been noted, increases in splitting tensile strength were observed. However, it must be noted that the results were significantly dependent on three factors: the orientation of fibres influenced by the direction of concreting, fibre content, and fibre length.

The tensile strength perpendicular to the direction of concreting was significantly lower than the strength parallel to the direction of concreting. In the case of tensile strength perpendicular to the direction of concreting (Figure 7a), the largest increase in strength, between 5% and 12%, was noted for CF-45 specimens with 45 mm long fibres. A similar trend was also observed for CF-35 samples with 35 mm long fibres—the strength increase varied from 5% to 10%. For CF-25 specimens with a fibre length of 25 mm, the strength increased by no more than 1%. Regarding the tensile strength parallel to the direction of concreting (Figure 7b), for CF-25 specimens it increased from 9% to 24%, for CF-35 specimens from 16% to 40%, and for CF-45 specimens from 17% to 66%, depending on the fibre content.

As previously mentioned, fibres tend to self-level during concrete casting and vibration; i.e., the fibres orient perpendicular to the direction of concreting. The orientation of fibres during testing has a crucial influence on the results and response of the sample. For this reason, the tensile strength of samples with fibres oriented perpendicular to the tensile plane (Figure 7b) was much higher than for samples with fibres arranged parallel to the tensile plane (Figure 7a). According to research [65], fibres oriented at an angle of 90° to 60° to the direction of concreting are effective in transferring tensile forces. Therefore, the correlation between the direction of concreting and the mode of loading should be taken into account in the design and construction of structural elements with FRC.

Furthermore, it was observed that the influence of fibre content on the tensile strength depends on the direction of concreting. The increase in tensile strength perpendicular to the direction of concreting for fibre dosages equal to 0.29% and 0.87% tended to decrease from about 10% to 6% for CF-35 and CF-45, while for CF-25 the tensile strength remained basically unchanged (Figure 7a). On the other hand, for splitting tensile tests parallel to the direction of concreting, a significant improvement in tensile strength was observed with increasing fibre content (Figure 7b). For samples with 0.29% of fibres 35 mm and 45 mm long, the tensile strength increased by about 17%, and for the dosage equal to 0.87% it increased by 40% for CF-35 and by 66% for CF-45. For samples with a fibre length of 25 mm, the increase in tensile strength was smaller, equal to 9% for a fibre content of 0.29% and 24% for a fibre content of 0.87%. For all fibre lengths, a very small enhancement in tensile strength was noted between specimens with 0.29% and 0.58% of fibres. On the contrary, a large increase in tensile strength was observed when fibre content increased from 0.58% to 0.87%.

In the study in [66], the compression and flexural performance of steel fibre-reinforced concrete (SFRC) with normal-strength (NSF) and high-strength (HSF) steel fibres was evaluated. Depending on the fibre content and length, the flexural tensile strength *f_L_* increased by up to 28% for NSF fibres and by up to 22.5% for HSF fibres. Additionally, the residual flexural tensile strength *f_R_* increased by up to 27% for NSF fibres and by up to 96% for HSF fibres. Comparing these results with those obtained for the splitting tensile strength of CFRC, it is important to note that the benefits of using rCF fibres are comparable. The 66% increase in tensile strength demonstrates the applicability of rCF fibres in cement composites.

Regardless of the direction of concreting and fibre content, a large effect of fibre length on tensile strength was observed. In all cases, the tensile strength increased with increasing fibre length. The effectiveness of fibres with lengths equal to 35 mm and 45 mm was similar, however, greater than that of 25 mm fibres. In other words, the tensile strength of specimens CF-35 and CF-45 was significantly higher than that of specimen CF-25. Increasing the length of the rCF increased the load-carrying capacity and post-peak deformation, where the energy released in the fracture process was absorbed by the fibres.

### 3.4. Brittleness

Furthermore, while evaluating the strength parameters of brittle materials, it is important to determine the reciprocal ratio between the splitting tensile strength *f_ct_* and the compressive strength *f*_cc_, called the Brittleness Index (*BI*), calculated according to Formula (3):

(3) $$BI=\frac{f_{ct}}{f_{cc}}$$

This is a measure of the material’s ability to suddenly fail under acting forces without disproportionate deformations preceding this failure.

**Figure 8:**
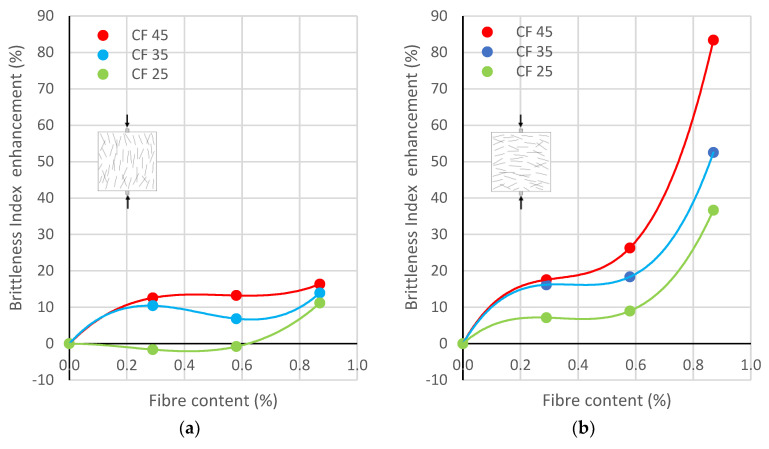
Brittleness Index enhancement vs. rCF content for different fibre lengths: (**a**) Perpendicular to the direction of concreting, (**b**) parallel to the direction of concreting.

The average values of the *BI* are provided in Table 3, and Figure 8 shows graphs of the relationship between Brittleness Index enhancement and fibre content for different lengths depending on the direction of concreting: perpendicular or parallel.

The ratio of tensile to compressive strength for plain concrete was equal to *f*_ct_/*f*_cc_ = 0.059, while for specimens with fibres, it was much higher. Analysing the data obtained, it can be concluded that the reference concrete had the highest brittleness, while the addition of fibres made the CFRCs more ductile. This was a result of the simultaneous increase in tensile strength and decrease in compressive strength, along with an increase in the amount and length of fibres. A large effect of the concreting direction was also noted. Namely, for the perpendicular direction (Figure 7a), the *BI* increased by a maximum of several percent for the highest fibre content of 0.87%. On the other hand, for the parallel direction, the *BI* enhancements were much larger and varied from 37% to 83% for the highest fibre content of 0.87%.

### 3.5. Fracture Energy

As a measure of the tensile strength of CFRC, the energy required to completely damage the specimens was calculated as the area under the load–vertical displacement curve. The area up to a given value of deflection is the total mechanical energy per unit volume consumed by the material in deforming it to that value. The fracture energy *FE* was calculated using the formula:

(4) $$FE=\sum {\bar{F}}_{i}∆d_{i}$$

where ${\bar{F}}_{i}$ is the average load at displacement increment Δ*d_i_*.

**Figure 9:**
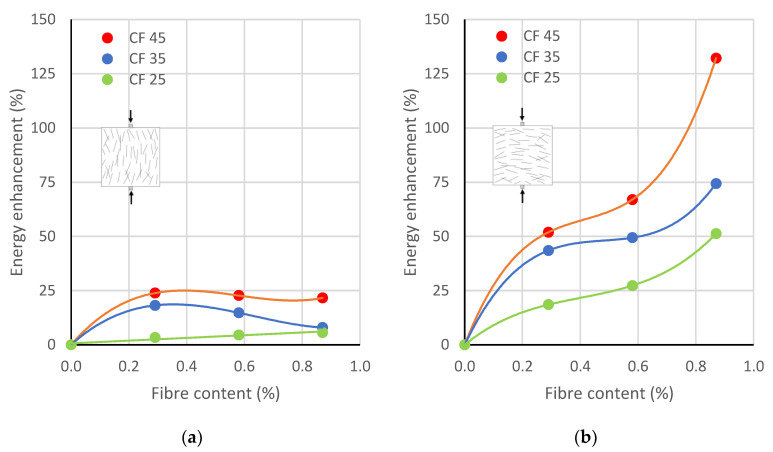
Energy enhancement vs. rCF content for different fibre lengths: (**a**) Perpendicular to the direction of concreting, (**b**) parallel to the direction of concreting.

The average calculated fracture energies are presented in Table 3. Moreover, charts of the relationship between fracture energy enhancement and fibre content for samples with different fibre lengths and split parallel and perpendicular to the direction of concreting are shown in Figure 9.

The curve trend for fracture energy for specimens split parallel to the direction of concreting (Figure 9b) was similar to that of the tensile strength (Figure 7b) and the Brittleness Index (Figure 8b). However, the enhancements in the amount of required fracture energy were greater than those in the tensile strength. For example, for a 0.87% dosage of fibres with lengths of 25 mm, 35 mm, and 45 mm, the tensile strength increased by 16.7%, 21.8%, and 66.0%, respectively, while the fracture energy rose by 51.9%, 66.9%, and 132.2%, respectively. The difference was two to three times. These variations pointed to the large ultimate deformations of the specimens at failure, which resulted from the greater bridging efficiency of longer fibres. The increase in the amount of required fracture energy was a consequence of the fibres’ ability to restrain the microcrack growth and to absorb energy by overcoming their pull-out.

### 3.6. Fracture Surface and Failure Mechanism

The effect of the rCF addition on the mechanical properties of CFRC was further examined based on the fracture surface images of the specimens after splitting. The analyses were conducted in order to determine the quantity and length of fibres that remained on the surfaces of the two halves of the specimens. Furthermore, their performance in transferring tensile forces was characterized by defining whether they ruptured or were pulled out from the concrete matrix.

**Figure 10:**
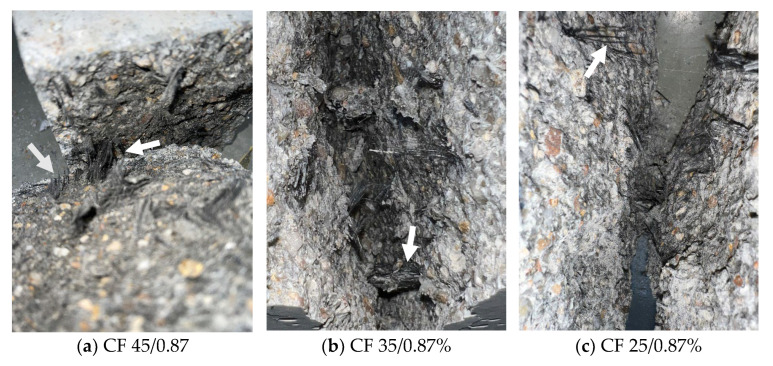
Fracture surfaces of chosen samples, tested perpendicular to the direction of concreting.

Figure 10 shows the fracture surfaces of three specimens split perpendicular to the direction of concreting for three fibre lengths corresponding to their highest dosage. About a dozen fibres with lengths ranging from a few to several millimetres were visible on the failure surfaces. The orientation angle of these fibres in relation to the cracking surface was very small and did not exceed 25°. Therefore, the crack bridging by the fibres was very limited, and hence only a slight increase in tensile strength, equal to less than 10%, was recorded (Figure 7a).

**Figure 11:**
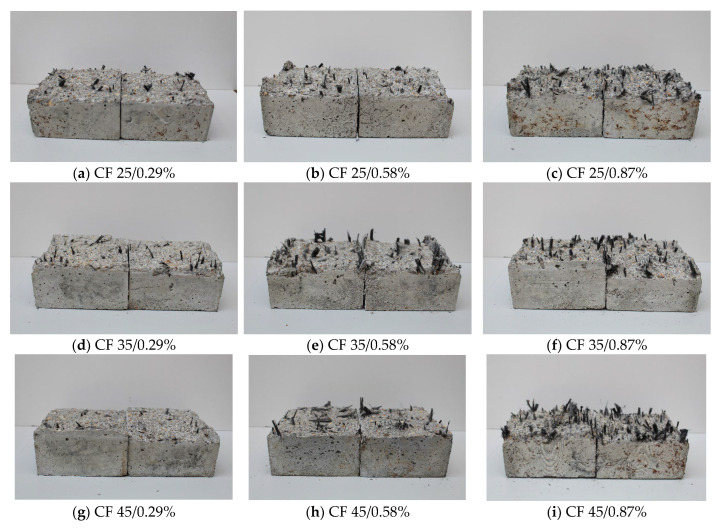
Fracture surfaces of chosen samples, tested parallel to the direction of concreting.

Pictures of the failure surfaces of tensile specimens tested parallel to the direction of concreting are shown in Figure 11.

It can be seen that the distribution of fibres on the failure surfaces was not uniform. Additionally, clusters of fibres were visible. This occurred because the carbon fibres were not thoroughly mixed or evenly distributed to create a uniform fibre network within the specimen, primarily due to the low consistency of the concrete mix. As expected, the smallest number of visible fibres was noted for samples with the lowest fibre content (0.29%), and the highest for specimens with the largest fibre content (0.87%). Moreover, the length of the fibres in the fracture plane depended on the original length of the fibre. The shortest fibres were seen in the cracked CF-25 specimens and the longest in the CF-45 specimens.

**Figure 12:**
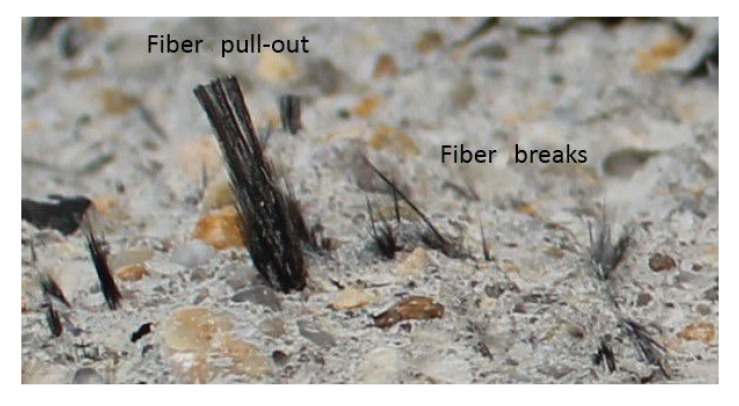
Fibre breakage and fibre pull-out in specimen CF 25.

Based on the analysis of fibres present on the fracture surfaces of the specimens, it can be concluded that some of them ruptured, and some of the fibres were pulled out from the concrete matrix as a result of debonding (Figure 12).

The failure mechanism of fibres had a major effect on tensile strength and, consequently, the splitting force required to destroy the specimen. It seems that the bridging effectiveness of fibres, understood as an increase in tensile strength, was greater when the fibres ruptured than when they were pulled out of the matrix. Furthermore, they also contributed to increasing the deformability.

Table 4 summarizes the results of the analysis of the fracture surfaces of the specimens split parallel to the concreting direction regarding the work of fibres that contributed to the transmission of tensile forces. The average number of fibres, their average and total length, and the mode of failure were determined.

It can be concluded that the number of fibres present on the failure surface was significantly dependent on fibre content and fibre length. The average fibre length *l* increased as their content and length increased. For CF-25 samples, the average fibre length varied between 8.4 mm and 11.0 mm, and for CF-35 samples it was between 10.5 mm and 13.8 mm, while for CF-45 it was between 15.8 mm and 16.9 mm. Similarly, the total fibre length *Σl* increased with fibre content and length. It ranged from 412 mm to 904 mm, 461 mm to 1063 mm, and 538 to 1285 mm for samples with fibre lengths of 25 mm, 35 mm, and 45 mm, respectively. The dependencies were different for the number of fibres *n* present on the fracture surfaces. Namely, with a decrease in used fibre length and content, the number of fibres *n* also decreased, i.e., for CF-25 (*n* = 49, 59, and 82 pcs), CF-35 (*n* = 44, 58, and 77 pcs), and CF-45 (*n* = 34, 50, and 76 pcs).

As previously mentioned, the failure mechanism of fibres present in the split specimens consisted of a combination of their rupture and pull-out from the matrix, with the pull-out being a dominant mode. Namely, the number of fibres pulled out from the matrix was greater than the number of fibres that ruptured.

## 4. Conclusions

The purpose of the experimental study was to understand the effect of the addition of recycled carbon fibres from wind turbine blades of different content and length on the concrete mechanical properties. Studies have shown that the direction of rCF orientation in relation to the applied tensile force has a decisive influence on the mechanical properties of CFRC, i.e., tensile strength, Brittleness Index, and fracture energy. The mechanical properties of CFRC split parallel to the direction of concreting were significantly better than those split perpendicular to the direction of concreting. The effectiveness of the fibres increased with increasing fibre content and length.

Moreover, it was concluded that the addition of rCFs slightly improved the mechanical properties of CFRC split perpendicular to the direction of concreting. Namely, the tensile strength was increased by a few to several percent up to a maximum of about 12%, the ductility—the Brittleness Index—grew by a maximum of 17%, and the fracture energy rose by a maximum of approximately 24%. On the other hand, the addition of rCFs significantly improved the mechanical properties of CFRC split parallel to the direction of concreting. It resulted in an increase in the splitting tensile strength from 10% to 66%, the Brittleness Index from 37% to 83%, and the fracture energy from 52% to 132%.

It was also concluded that the results obtained for the mechanical properties of CFRC were closely related to the behaviour and characteristics of the rCF, which were visible on the fracture surfaces of the tensile specimens. The number of fibres, their length, and the mode of fibre failure strongly influenced the tensile strength and hence the Brittleness Index and fracture energy. The obtained improvements in the concrete properties after the addition of carbon fibres were a result of their ability to resist the growth of microcracks and absorb fracture energy. The study showed that a combination of rCF pull-out and rupture in the transmission of tensile forces in the CFRC occurred. Namely, some of the fibres ruptured, and some of them were pulled out from the matrix, with the predominant failure mode being the pull-out.

Additionally, the results of the study showed that reinforcing concrete with rCFs of different content and lengths is effective from the mechanical point of view, on the condition that the correct direction of fibre orientation is assured. However, this strongly depends on the direction of concreting. It must also be highlighted that the increases in tensile strength obtained within this experimental campaign are in line with other studies from the literature review.

Finally, it is relevant to note that the research presented in this article is preliminary and cannot be considered an exhaustive work providing a full understanding of the effects of rCFs on the mechanical behaviour of concrete. Further research needs to be performed, taking into consideration the conclusions drawn from the performed tests. Namely, to improve the mechanical properties of CFRCs, the addition of fibres with higher roughness can be tested to ensure better interaction with the concrete matrix. Furthermore, plasticizers should be used to improve the workability of the concrete mixture and thus the efficiency of compaction. It will also lead to a more uniform fibre distribution and prevent cluster formation. Besides this, it is also advisable to test higher fibre contents and different fibre lengths. In addition, long-term tests should be carried out in order to characterize the rheological properties of CRFC and its environmental impact.

To sum up, the research results presented within the scope of this article, together with others available in the literature, can help to assess the feasibility of using recycled wind turbine blade waste in the construction industry. Undoubtedly, applying these research findings can contribute to lowering CO_2_ emissions and preserving natural resources.

## Figures and Tables

**Table 1:** Composition of concrete mixes in kg/m^3^.

Mix ID	Cement	Water	Aggregate 0/2 mm	Aggregate 2/8 mm	rCF Fibre
C-0	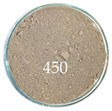	270	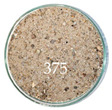	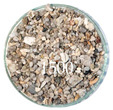	0
CF-5					5.0
CF-10					10.0
CF-15					15.0

**Table 2:** Workability of concrete mixes.

Series ID	C-0	CF-25			CF-35			CF-45		
rCF length (mm)	-	25			35			45		
rCF dosage (%)	0	0.29	0.58	0.87	0.29	0.58	0.87	0.29	0.58	0.87
Slump (mm)	140	30	20	10	30	20	10	30	20	10
Consistency class	S3	S1			S1			S1		

**Table 3:** Average values obtained from the tests.

Series ID	rCF (%)	$f_{cc}$(MPa)	$f_{ct,⊥}$ (MPa)	$f_{ct,∥}$ (MPa)	*BI* (−)		${FE}_{⊥}$ (J)	$FE_{∥}$ (J)
					$f_{ct,⊥}/f_{cc}$	$f_{ct,∥}/f_{cc}$		
C-0	0	44.4 (1.5)	2.62 (0.16)		0.059		88 (5)	
CF-25	0.29	45.3 (1.6)	2.63 (0.15)	2.86 (0.18)	0.058	0.063	91 (9)	109 (5)
	0.58	45.1 (1.3)	2.64 (0.21)	2.90 (0.11)	0.059	0.064	92 (10)	122 (10)
	0.87	40.4 (1.9)	2.65 (0.17)	3.26 (0.18)	0.066	0.081	99 (8)	150 (9)
CF-35	0.29	44.2 (1.3)	2.88 (0.19)	3.03 (0.15)	0.065	0.069	104 (12)	132 (8)
	0.58	44.4 (1.7)	2.80 (0.11)	3.10 (0.20)	0.063	0.070	102 (10)	143 (7)
	0.87	40.9 (1.5)	2.75 (0.18)	3.68 (0.12)	0.067	0.090	95 (9)	173 (9)
CF-45	0.29	44.1 (1.1)	2.93 (0.15)	3.06 (0.20)	0.066	0.069	108 (11)	150 (10)
	0.58	42.8 (1.5)	2.86 (0.19)	3.19 (0.17)	0.067	0.075	109 (8)	173 (12)
	0.87	40.2 (1.7)	2.76 (0.21)	4.35 (0.28)	0.069	0.108	107 (10)	231 (13)

Note: The value in parentheses represents the standard deviation of each sample set. The number of samples was 3.

**Table 4:** Fibres on the fracture surfaces for samples tested parallel to the direction of concreting.

Series ID	CF 25			CF 35			CF 45		
rCF (%)	0.29	0.58	0.87	0.29	0.58	0.87	0.29	0.58	0.87
*l* (mm)	8.4	10.1	11.0	10.5	13.6	13.8	15.8	16.3	16.9
Σ *l* (mm)	412	596	904	461	796	1063	538	814	1285
*n* (pc)	49	59	82	44	58	77	34	50	76

Notations: rCF is fibre content, *l* is average fibre length, Σ *l* is total fibre length, *n* is number of fibres.

## Data Availability

The raw data supporting the conclusions of this article will be made available by the authors on request.
